# C,N co-doped TiO_2_ hollow nanofibers coated stainless steel meshes for oil/water separation and visible light-driven degradation of pollutants

**DOI:** 10.1038/s41598-023-28992-4

**Published:** 2023-04-07

**Authors:** Chunyu Wang, Yingze Liu, Hao Han, Desheng Wang, Jieyi Chen, Renzhi Zhang, Shixiang Zuo, Chao Yao, Jian Kang, Haoguan Gui

**Affiliations:** 1 State Key Laboratory of NBC Protection for Civilian, Beijing, 102205 China; 2 Department of Chemical Engineering, Tsinghua University, Beijing, 100084 China; 3 School of Petrochemical Engineering, Changzhou University, Changzhou, 213164 China; 4 Jiangsu Key Laboratory of Advanced Catalytic Materials and Technology, Advanced Catalysis and Green Manufacturing Collaborative Innovation Center, Changzhou University, Changzhou, 213164 China

**Keywords:** Pollution remediation, Catalysis, Photocatalysis

## Abstract

Complex pollutants are discharging and accumulating in rivers and oceans, requiring a coupled strategy to resolve pollutants efficiently. A novel method is proposed to treat multiple pollutants with C,N co-doped TiO_2_ hollow nanofibers coated stainless steel meshes which can realize efficient oil/water separation and visible light-drove dyes photodegradation. The poly(divinylbenzene-*co*-vinylbenzene chloride), P(DVB-*co*-VBC), nanofibers are generated by precipitate cationic polymerization on the mesh framework, following with quaternization by triethylamine for N doping. Then, TiO_2_ is coated on the polymeric nanofibers via in-situ sol–gel process of tetrabutyl titanate. The functional mesh coated with C,N co-doped TiO_2_ hollow nanofibers is obtained after calcination under nitrogen atmosphere. The resultant mesh demonstrates superhydrophilic/underwater superoleophobic property which is promising in oil/water separation. More importantly, the C,N co-doped TiO_2_ hollow nanofibers endow the mesh with high photodegradation ability to dyes under visible light. This work draws an affordable but high-performance multifunctional mesh for potential applications in wastewater treatment.

## Introduction

The separation and treatment of wastewater with complex pollutants are always an intractable problem in industry and environmental science. The spilled oil, discharged from textiles, mining, foods, petroleum, metal/steel industries, and ocean shipping, has caused serious worldwide ecological disasters^1–4^. Cleanup technologies for the spilled oil are urgently needed, attracting researchers to develop efficient strategies for the oily wastewater treatment. The physical oil/water separation, based on the superwetting materials, has been widely studied due to the low energy consuming and the high efficiency^5–7^. Superhydrophobic/superoleophilic filters or absorbents are most used for the oil/water separation by damming the water and passing or absorbing the oil^8–12^. However, these superhydrophobic/superoleophilic surfaces are easy to be fouled and blocked once separating highly viscous oils, e.g., crude oil, from the oily wastewater.

To solve this problem, two main strategies have been proposed. Some researchers attempted to reduce the viscosity of the surrounding crude oil with an external heating source, e.g., joule-heating^13–15^, photothermal conversion^14,16–18^, electromagnetic induction^19,20^. The other strategy was the fabrication of superhydrophilic/underwater superoleophobic membranes which have been deserved more attention^21^. Water can pass through the superhydrophilic/underwater superoleophobic membrane, but oil is repelled, avoiding the polluting of the membrane. To achieve the superhydrophilic/underwater superoleophobic surface, the membrane or framework was usually coated with hydrogel networks^22,23^, polyelectrolyte chains^24,25^, zwitterionic polymers^26,27^, hydrophilic polysaccharides^28,29^, etc. As most inorganic nanoparticles containing hydrophilic groups, they could be coated or in situ grew on the membrane or framework to fabricate the superhydrophilic/underwater superoleophobic surface. For example, the inorganic composite membranes with superhydrophilic/underwater superoleophobic surface have been prepared from metallic oxide nanoparticles (e.g., SiO_2_^30,31^, ZnO^32^, TiO_2_^33–35^, NiO^36^, WO_3_@Cu(OH)_2_^37^, ZnO@Cu_2_O^38^, CuWO_4_@Cu_2_O^39^), metal nanoparticles (e.g., Ag^40,41^, Ni^42^, Cu^43^), Zeolite^44^, MXene^35,45^ and MOF^46,47^. Moreover, some inorganic functional components exhibit photocatalytic activity, realizing the purification of wastewater with complex pollutants.

As above-mentioned, the components of wastewater are complex, desiderating multi-step treatment. The water-soluble pollutants cannot be easily disposed with the physical separation. Therefore, the multifunctional membranes with inorganic nanoparticles can be came in handy. Among them, superhydrophilic/underwater superoleophobic membranes loaded with photocatalyst can not only separate oil/water mixture but also realize degradation of pollutants such as dyes in water, which has broad application prospects^48,49^. However, due to the intrinsic wide band gap of most inorganic nanoparticles, they realized photocatalytic degradation of the water-soluble pollutants only under UV light irradiation^38^. It is challenging to realize photodegradation of pollutants under visible light irradiation. Alternatively, photodegradation under visible light irradiation have been achieved with low band gap nanoparticles (e.g., CuWO_4_@Cu_2_O^39^) or lowering the band gap of metal oxide semiconductor with highly metallic conductive carriers (e.g., graphene oxide^34,50^, carbon nitride^51,52^, MXene^35,45,53,54^ and metal sulfide^55–58^). However, these strategies generally need heavy metal suffer from toxicity and complex process. To achieve visible light photocatalyst with low price, metal oxide semiconductors, especially TiO_2_ based nanomaterials, have been doped with hybrid atoms for low band gap^59–63^. The C,N co-doped TiO_2_ exhibits a peculiarly better visible light catalytic activity^64–66^. It should be feasible to prepare TiO_2_ based membranes with visible light photocatalytic activity and superwetting properties simultaneously.

**Figure 1:**
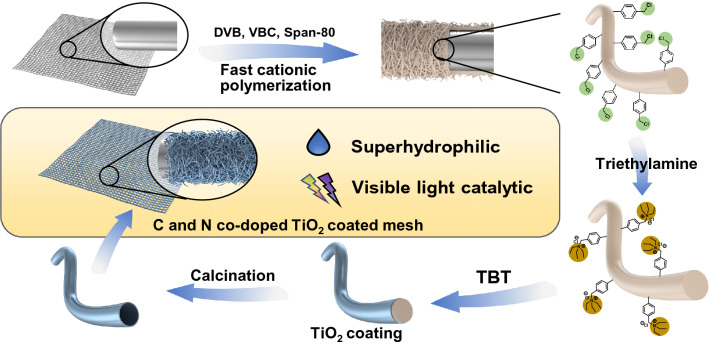
Schematic preparation of the C,N co-doped TiO_2_ hollow nanofibers coated mesh.

Herein, a C,N co-doped TiO_2_ hollow nanofibers coated stainless steel mesh with superhydrophilic/underwater superoleophobic surface and excellent visible light photocatalytic activity is developed as illustrated in Fig. 1. First of all, the polymeric poly(divinylbenzene-*co*-vinylbenzene chloride), P(DVB-*co*-VBC), nanofibers coated mesh was prepared by the precipitate cationic polymerization following with our previous work^11,26,67,68^. Then, the N element and cationic group were introduced through the quaternization of triethylamine with benzyl chloride functional group in the PVBC segment. After that, core–shell P(DVB-*co*-VBC)@TiO_2_ composite nanofibers coated mesh was prepared with in-situ sol–gel process of tetrabutyl titanate. At last, the C,N co-doped TiO_2_ hollow nanofibers coated mesh was obtained by calcination under nitrogen atmosphere. The resultant composite mesh exhibited excellent superhydrophilic/underwater superhydrophobic surface which could separate oil/water mixture promptly. Furthermore, the composite mesh could realize dye degradation under visible light irradiation with high efficiency, demonstrating the key role of C,N co-doped TiO_2_ hollow nanofibers. The C,N co-doped TiO_2_ hollow nanofibers coated mesh is a prospective candidate for the integrative treatment of complex wastewater.

## Experimental section

### Materials

Divinylbenzene (DVB, 80%, Tokyo Chemical Industry) and vinylbenzene chloride (VBC, 99.9%, Tokyo Chemical Industry) were purified through neutral aluminum oxide column to remove the inhibitor. Tetrabutyl titanate (TBT, 98%, Tokyo Chemical Industry), triethylene amine (99%, Shanghai Macklin Biochemical Technology), boron trifluoride diethyl etherate (BFEE, Tokyo Chemical Industry), *n*-heptane (99.9%, Shanghai Titan Technology), polysorbitan monooleate (Span-80, Sinopharm Chemical Reagent Beijing), anhydrous ethanol (Shanghai Titan Technology) and stainless steel meshes (2000 mesh, Shanghai Titan Technology) were used as received.

### Preparation of P(DVB-*co*-VBC) nanofibers coated mesh

The P(DVB-*co*-VBC) nanofibers coated stainless steel mesh was prepared following our previous work with a few alterations^11,26,67,68^. Briefly, an example stainless steel mesh (2 cm × 2 cm) was immersed in 50 mL of *n*-heptane containing 10 mg of Span-80 as the surfactant. After dropping 20 μL of BFEE, the mixture was ultrasonicated for 30 s to form the BFEE dispersion. The monomer mixture containing 200 μL of DVB and 200 μL of VBC in 10 mL of *n*-heptane was fed to the BFEE mixture under ultrasonication for the precipitate cationic polymerization. After polymerization for 90 s, 1.0 mL of ethanol was added to terminate the polymerization. The P(DVB-*co*-VBC) nanofibers coated mesh was prepared.

### Modification of P(DVB-*co*-VBC) nanofibers coated mesh

The P(DVB-*co*-VBC) nanofibers were in-situ modified with triethylamine which occurs quaternization with PVBC segments. The quaternary ammonium salt modified P(DVB-*co*-VBC) nanofibers were denoted as P(DVB-CH_2_N^+^Cl^−^) nanofibers. Briefly, P(DVB-*co*-VBC) nanofibers coated mesh (2 cm × 2 cm) was immersed in 10 ml of ethanol. Then, 2 mL of triethylamine was added to the solution. The reaction was performed at room temperature for 24 h. P(DVB-CH_2_N^+^Cl^−^) nanofibers coated mesh was collected after rinsing with water and ethanol for three times and drying under ambient atmosphere.

### Preparation of P(DVB-CH_2_N^+^Cl^−^)@TiO_2_ nanofibers coated mesh

The P(DVB-CH_2_N^+^Cl^−^)@TiO_2_ nanofibers coated mesh was prepared by in-situ sol–gel process of TBT on P(DVB-CH_2_N^+^Cl^−^) coated mesh. The P(DVB-CH_2_N^+^Cl^−^) coated mesh was immersed in 40 mL of ethanol. Then, 200 μL of TBT was added to the solution. The reaction was performed at 70 °C for 12 h. After adding 2 mL of water into the solution, the P(DVB-CH_2_N^+^Cl^−^)@TiO_2_ nanofibers coated mesh was obtained for another 12 h of reaction, rinsing with ethanol and water for three times.

### Preparation of C,N co-doped TiO_2_ hollow nanofibers coated mesh

The C,N co-doped TiO_2_ hollow nanofibers coated mesh was prepared by calcination of P(DVB-CH_2_N^+^Cl^−^)@TiO_2_ nanofibers coated mesh at 450 °C for 2 h under nitrogen atmosphere. The resultant C,N co-doped TiO_2_ hollow nanofibers coated mesh was denoted as TN450 coated mesh.

### Characterization

Scanning electron microscope (SEM) measurement was performed on a JEOL 7900F equipped with an energy dispersive X-ray (EDX) analyzer. The samples for SEM observation were ambient dried and sputtered with Pt in vacuum. X-ray photoelectron spectroscopy (XPS) measurement was performed on an X-ray photoelectron spectrometer (Thermo scientific, ESCALAB 250XI) with Al Kα radiation. Contact angle measurements of water and oil were performed on video optical contact angle measuring device (OCAH200) at ambient temperature. X-ray diffraction (XRD) was performed on a X'Pert PRO MPD diffractometer at room temperature. UV–vis absorbance spectroscopy and UV–vis diffuse reflectance spectroscopy were recorded on a UV–Vis spectroscope (Agilent Cary Series).

### Photocatalysis experiment

The photocatalytic experiment employed a 300 W xenon lamp (CEL-HXF300/CEL-HXUV300, Beijing Zhongjiaojinyuan Technology Co. Ltd.) as the light source, which can emit UV or visible light with optical filters. The UV emission wavelength and visible light emission wavelength are in the range of 200–420 nm and 420–800 nm, respectively. The methylene blue concentration of the standard solution was 15 mg/L in the catalytic experiment. A piece of photocatalytic mesh (2 cm × 2 cm) was immersed in a beaker containing 50 mL of methylene blue solution. The beaker was placed in a water bath with the constant temperature at 25 °C. The liquid level was kept at 15 cm away from the light source. The residual concentration of methylene blue was recorded with a UV–Vis spectrophotometer as a function of time once turning on the switch.

## Results and discussion

**Figure 2:**
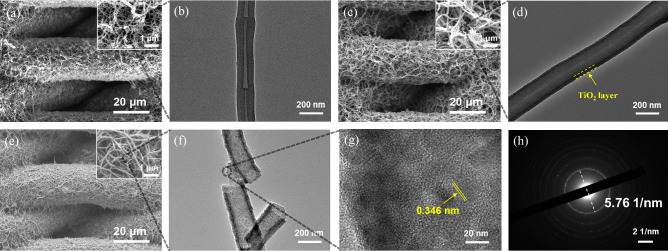
(**a**) SEM image of P(DVB-CH_2_N^+^Cl^−^) nanofibers coated mesh; (**b**) TEM image of P(DVB-CH_2_N^+^Cl^−^) nanofiber; (**c**) SEM image of P(DVB-CH_2_N^+^Cl^−^)@TiO_2_ nanofibers coated mesh; (**d**) TEM image of P(DVB-CH_2_N^+^Cl^−^)@TiO_2_ nanofiber; (**e**) SEM image of TN450 coated mesh; (**f**) TEM image of TiO_2_ hollow nanofiber; (**g**) high resolution TEM image of TiO_2_ hollow nanofiber; (**h**) the corresponding selected-area electron diffraction pattern.

An example P(DVB-*co*-VBC) nanofibers coated mesh was prepared by precipitate cationic polymerization of DVB and VBC in the presence of span-80 as our previous work^26^. The SEM image of pristine stainless steel mesh (size of 2000 mesh) shows the wires with smooth surface (Fig. S1a). After polymerization of P(DVB-*co*-VBC), the stainless steel wires are covered with a layer of polymeric nanofibers (Fig. S1b). The P(DVB-CH_2_N^+^Cl^−^) coated mesh with nanofibrous structure was obtained after in-situ quaternization of triethylamine by the PVBC segments. The resultant P(DVB-CH_2_N^+^Cl^−^) nanofibers exhibit the same morphology as the original P(DVB-*co*-VBC) nanofibers on the coated mesh (Fig. 2a). TEM image proposes the structure of a single P(DVB-CH_2_N^+^Cl^−^) nanofiber, showing a bamboo-like structure (Fig. 2b). After in-situ sol–gel process of TBT, the P(DVB-*co*-VBC)@TiO_2_ nanofibers coated mesh is prepared with similar morphology (Fig. 2c), while TEM image shows the nanofiber with a core–shell structure (Fig. 2d). EDX mapping image shows the presence of C, Ti, N, Cl elements which C and Ti dominate the composition (Fig. S2a). TN450 coated mesh was prepared after calcination at 450 °C under nitrogen atmosphere. As shown in Fig. 2e, the resultant TN450 coated mesh exhibits nanofibrous network on the wires surface, which is crucial to the superwettability property. EDX mapping image of TN450 coated mesh shows that C, Ti, N elements can be seen on the surface while Cl element disappears (Fig. S2b). After the calcination process, the C and N element exist with significant decrease in the content. TEM image shows the fractured TiO_2_ nanofiber with a typical hollow structure which is contributed by burning the polymeric core and remaining the inorganic shell under calcination (Fig. 2f). High resolution TEM image shows distinct lattice fringes with a major interplanar spacing of 0.346 nm (Fig. 2g), consistent with the anatase (101) interplanar spacing. The results match the corresponding selected-area electron diffraction pattern (Fig. 2h).

**Figure 3:**
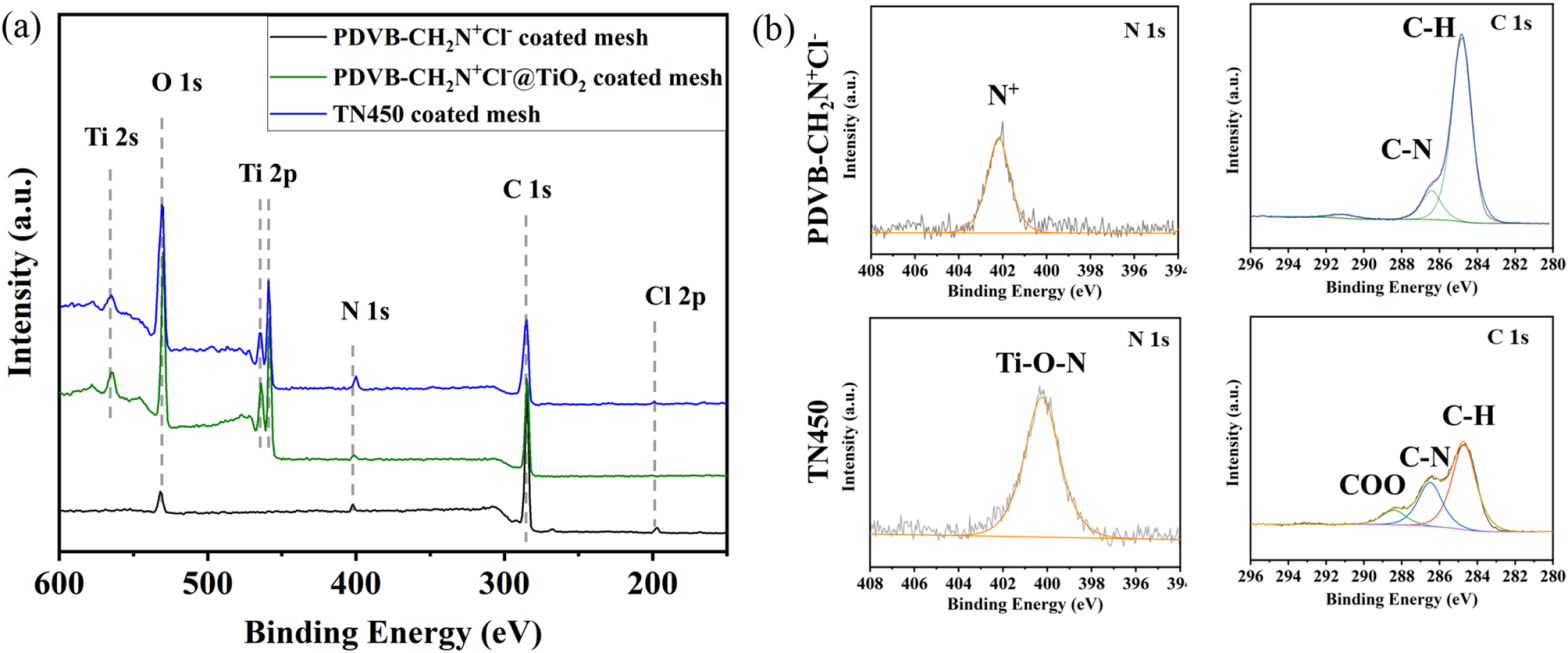
(**a**) XPS spectra of PDVB-CH_2_N^+^Cl^−^ coated mesh, PDVB-CH_2_N^+^Cl^−^@TiO_2_ coated mesh and TN450 coated mesh; (**b**) the deconvoluted C 1*s* and N 1*s* spectra of PDVB-CH_2_N^+^Cl^−^ coated mesh and TN450 coated mesh.

The surface composition of PDVB-CH_2_N^+^Cl^−^ coated mesh, PDVB-CH_2_N^+^Cl^−^@TiO_2_ coated mesh and TN450 coated mesh were characterized by XPS (Fig. 3a). The XPS spectrum of PDVB-CH_2_N^+^Cl^−^ composite metal mesh shows the existence of emission peaks for C 1*s* (284.8 eV), N 1*s* (402.5 eV), Cl 2*p* (197.0 eV). The presence of O 1*s* (532.0 eV) is the result of the surfactant Span-80 participated in the polymerization process at the interface^67,69^. The deconvoluted peak of N 1*s* (402.5 eV) is attributed to the positively charged amino groups (Fig. 3b). The structure of PDVB-CH_2_N^+^Cl^−^ can be further ascertained by the presence of C–N and C–H species on the deconvoluted C 1*s* spectrum. After in-situ growth of TiO_2_, new peaks assign to Ti 2*s* and Ti 2*p* emerge. The intensity of O 1*s* peak is greatly enhanced which associated with the generation of TiO_2_. The intensity of N 1*s* peak is reduced, indicating that PDVB-CH_2_N^+^Cl^−^ surface has been coated with a layer of TiO_2_. After calcination in nitrogen atmosphere at 450 °C, the emission peak of N element in TN450 is shifted to 400.2 eV, signifying that the nitrogen is in the Ti–O–N site^59,70^. Meanwhile, the peak intensity is significantly enhanced. The reason can be attributed to the migration of N element from core to the shell during the calcination process. The deconvoluted peak of C 1*s* shows a new peak at 288.4 eV which belongs to C=O, indicating the partial oxidization of the polymer during the calcination process. Taken together, these results demonstrate the successful doping of N and C elements on TN450 coated mesh.

**Figure 4:**
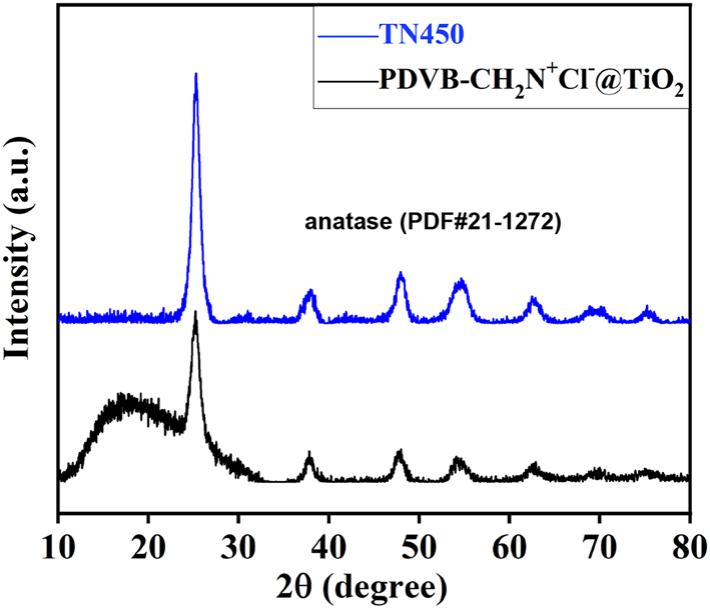
The XRD spectrum of PDVB-CH_2_N^+^Cl^−^@TiO_2_ and TN450.

The crystal form of TiO_2_ before and after calcination were characterized by XRD, as shown in Fig. 4. Before calcination, the XRD pattern of PDVB-CH_2_N^+^Cl^−^@TiO_2_ coated mesh has a big bulge which is attributed to the amorphous polymer. The diffraction peaks at 25.3° (101), 37.8° (004), 48.0° (200), 53.9° (105), 55.0° (211), 62.7° (204), 68.8° (116), 70.3° (220) and 75.0° (215) are assigned to the crystal planes of anatase (the ASTM Card, PDF#21-1272). After calcination, the bulge is completely disappeared due to the burning polymeric component. The XRD pattern only displays the anatase structure, demonstrating the crystal form of TiO_2_ changed little.

**Figure 5:**
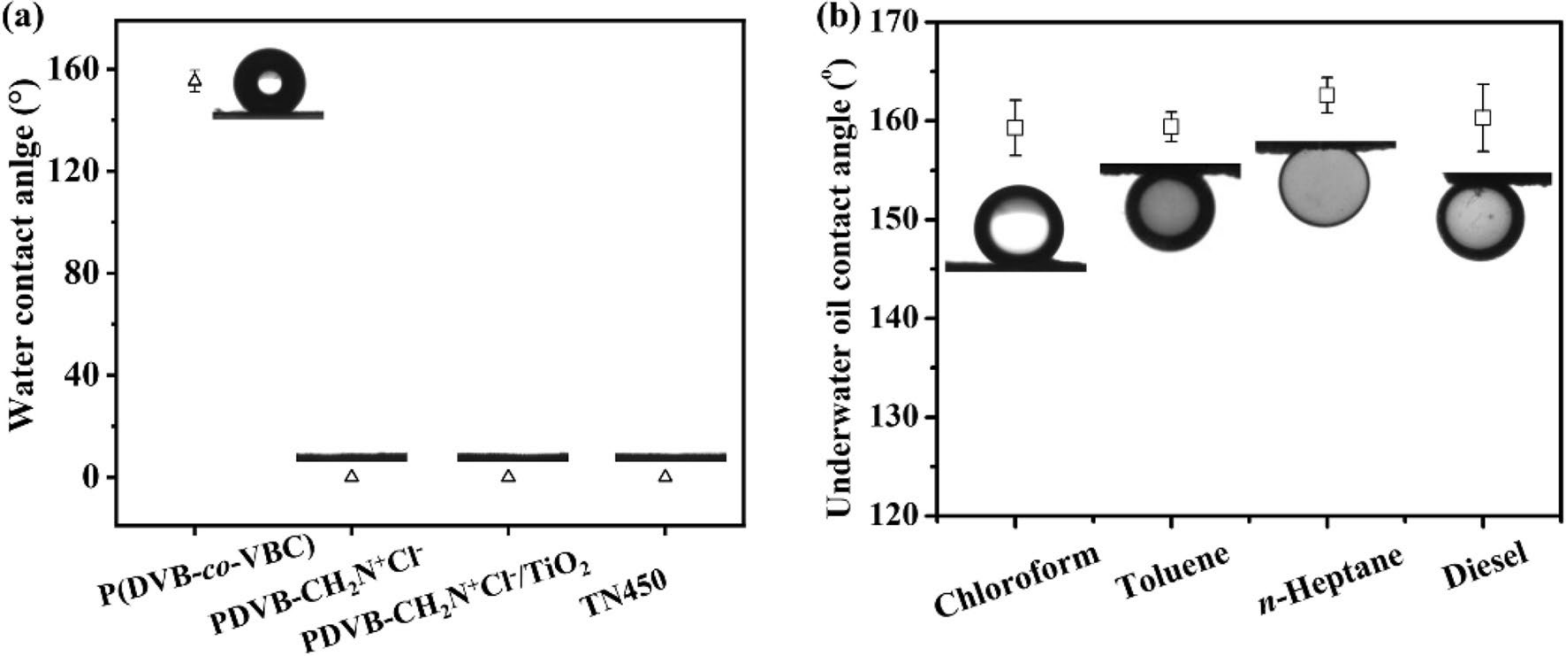
(**a**) The water contact angle versus surface chemistry of the coated mesh; (**b**) Under water oil contact angle of TN450 coated mesh (model oil: chloroform, toluene, n-heptane, and diesel).

The surface wettability varies with surface chemistry (Fig. 5a). The P(DVB-*co*-VBC) nanofibers coated mesh exhibits superhydrophobic property with a water contact angle (WCA) over 155° which is the same as our previous work^26^. After quaternization, the coated mesh becomes superhydrophilic (WCA = 0°) due to the presence of a large number of hydrophilic quaternary ammonium salt functional groups on the polymer chains, forming a long-range hydrophilic attraction with water^71^. After in-situ sol–gel process with TBT, the nanofiber surface has been covered with TiO_2_, resulting the WCA at 0° due to the high surface energy of inorganic materials. The rough and nanofibrous structure can be completely maintained after further sintering at 450 °C, which is crucial to the superhydrophilic property of TN450 coated mesh. The WCA of TN450 coated mesh is still at 0°, confirming a large number of hydrophilic Ti–OH chemical bonds remained after calcination. The TN450 coated mesh, more importantly, exhibits underwater superoleophobic properties with an underwater contact angle up to 159.3 ± 2.8°, 159.4 ± 1.5°, 162.6 ± 1.8° and 160.3 ± 3.4° to chloroform, toluene, *n*-heptane, and diesel, respectively (Fig. 5b). The composition of PDVB-CH_2_N^+^Cl^−^ and TiO_2_ is important to the special wettability of the TN450 coated mesh. As a comparison, the PDVB-CH_2_N^+^Cl^−^ coated mesh without TiO_2_ was calcinated under nitrogen at 450 °C for 2 h. The nanofibrous structure of resultant mesh is partially fused, resulting in the disappearance of rough structure and special wettability (Fig. S3).

**Figure 6:**
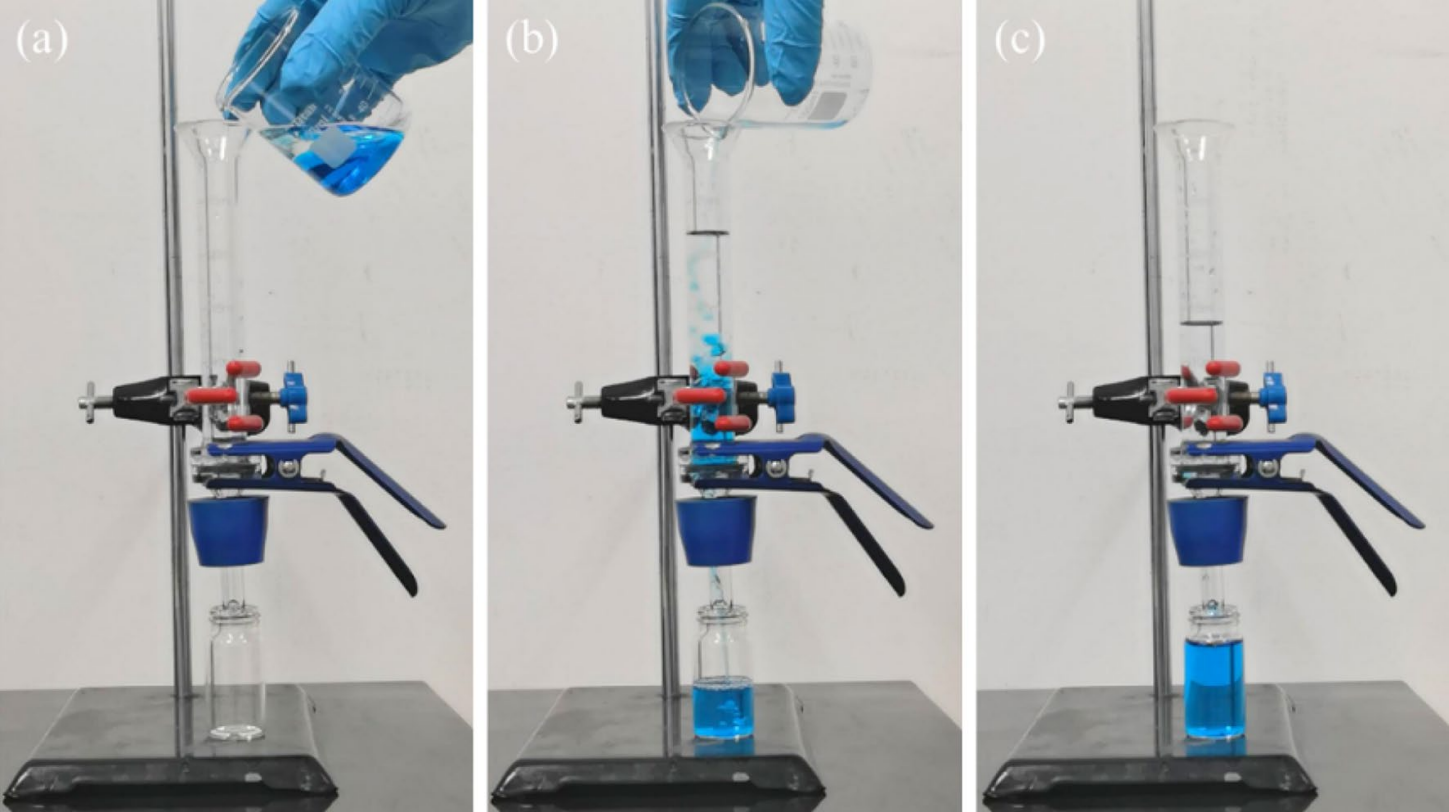
(**a**–**c**) Separation of *n*-heptane/dyed water mixture.

The superhydrophilic/underwater superoleophobic properties of TN450 coated mesh can effectively prevent meshes to be polluted by oils but allow the water to pass through meshes during the oil/water separation. A model wastewater was simulated by the mixture of *n*-heptane and dyed water which contains 15 mg/L of methylene blue. Since the TiO_2_ surface can be also wettable by oil, the TN450 coated mesh should be prewetted by water before sandwiched between the fixtures for oil/water separation (Fig. 6a). The model wastewater was poured into the glass tube from above. The aqueous phase quickly flowed through the filter mesh, while the colorless oil phase was trapped (Fig. 6b). Finally, the dyed aqueous phase was completely passed through the filter, while the colorless oil phase was completely trapped (Fig. 6c). Thus, oil/water separation can be achieved owe to the superhydrophilic rough surface of the meshes for the possibly trapping the water molecules, decreasing the oil adhesion under the “Cassie-Baxter” surface^72^. Moreover, the TN450 coated mesh possesses a quite high proof pressure of the example oil, *n*-heptane, and a high flux for water, which are similar as our previous works^11,26^.

After oil/water separation, the TN450 coated mesh was collected and employed for photodegradation of organic pollutants in the separated water under UV or/and visible light irradiation. Firstly, the standard methylene blue solution with different concentration were prepared. The main absorption peak of methylene blue is located at 664 nm (Fig. S4a). According to the Lambert–Beer law, the concentration of methylene blue is proportional with the peak intensity. The standard curve is plotted with a correlation coefficient of 0.999 (Fig. S4b). The residual concentration of methylene blue can be calculated according to the intensity of the absorption peak.

**Figure 7:**
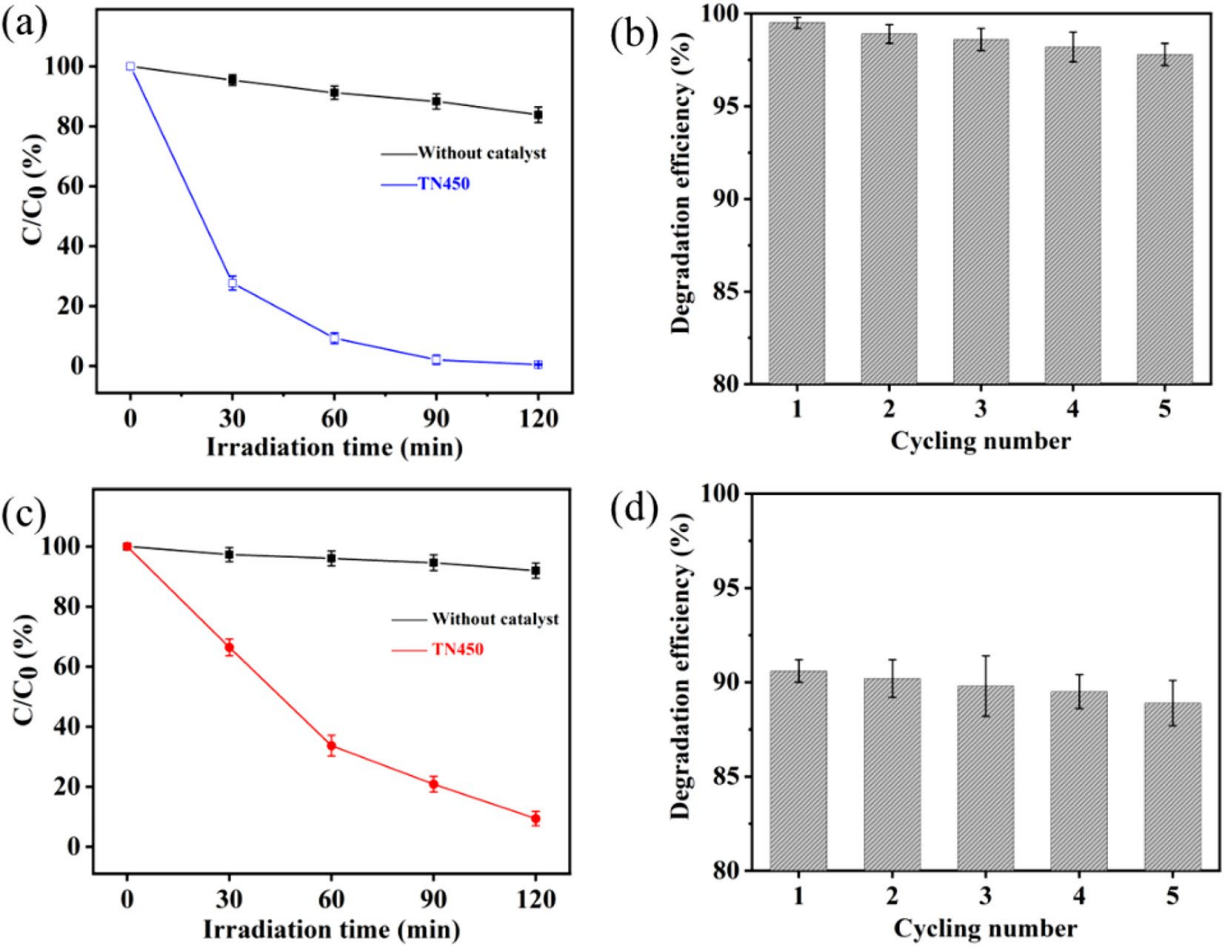
(**a**) The photocatalytic degradation of methylene blue with TN450 coated mesh versus irradiation time under UV light; (**b**) the recyclable experiment of methylene blue degradation under UV light; (**c**) the photocatalytic degradation of methylene blue with TN450 coated mesh versus irradiation time under visible light; (**d**) the recyclable experiment of methylene blue degradation under visible light.

Photocatalytic performance of the TN450 coated mesh was characterized with the photodegradation of methylene blue under UV light firstly. The TN450 coated mesh (2 × 2 cm) was immersed in 50 mL of separated wastewater containing the methylene blue with a concentration of 15 mg/L. The self-degradation of methylene blue was processed under the ultraviolet light as a function of time (Fig. 7a). After 120 min of UV light irradiation, the residual content of methylene blue is at 83.8 ± 2.6%, corresponding to a self-degradation efficiency of 16.2 ± 2.6%. In the case of TN450 coated mesh photocatalysis, the methylene blue is rapidly degraded with a residual content of only 27.7 ± 2.3% at 30 min, identifying the degradation efficiency of 72.3 ± 2.3%. When the photodegradation is prolonged to 120 min. the residual content of methylene blue is only 0.5 ± 0.2%, confirming the methylene blue is almost fully degraded. Besides the photocatalytic activity, the photocatalytic stability and recyclability of the TN450 coated mesh was also processed. After five cycles of photocatalysis experiments, the degradation efficiency of methylene blue is kept at a high level of about 98% under 120 min of UV light irradiation (Fig. 7b). These results show that the TN450 coated mesh has an excellent photocatalytic ability, good photocatalytic stability, and recyclability under UV light.

Furthermore, the photocatalytic degradation of methylene blue was also possessed with the TN450 coated mesh under visible light irradiation (Fig. 7c). Methylene blue is more stable under visible light with a residual content of 92.0 ± 2.5% and a degradation efficiency of 8.0 ± 2.5% after 120 min irradiation. Nevertheless, the residual content of methylene blue is at only 9.4 ± 2.4% when photocatalyzed by the TN450 coated mesh under 120 min irradiation of visible light. It confirmed that the degradation efficiency of methylene blue can be as high as 90.6 ± 2.4%, showing excellent visible light photocatalytic ability of the TN450 coated mesh. Moreover, the photocatalytic stability and recyclability of the TN450 coated mesh was also processed under the visible light irradiation (Fig. 7d). The results exhibit the TN450 coated mesh with a better photocatalytic stability under the visible light irradiation, retaining the similar degradation efficiency of methylene blue at around 90% for each cycle of the photodegradation experiments. Hence, the TN450 coated mesh is a promising candidate in the photocatalytic degradation of organic pollutants under the solar light irradiation.

**Figure 8:**
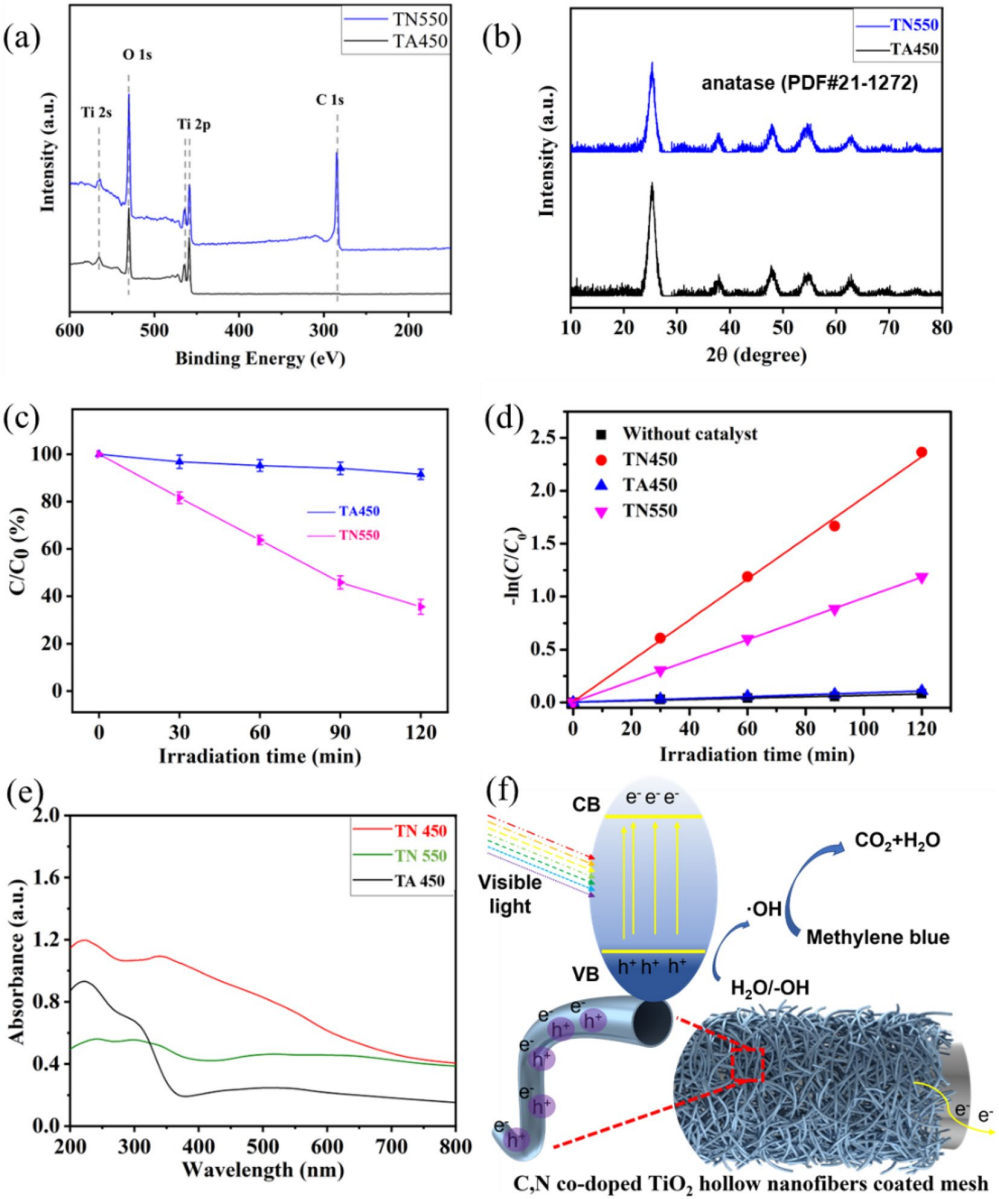
(**a**) The XPS spectrum of TA450 coated mesh and TN550 coated mesh; (**b**) The XRD spectrum of TN550 and TA450; (**c**) the photocatalytic performance of TA450 and TN550 coated mesh under visible light; (**d**) variations of − ln(*C*/*C*_0_) versus irradiation time with neat mesh, TN450, TA450, and TN550 coated meshes under visible light; (**e**) the UV–Vis diffuse reflectance spectrum of TN450, TN550 and TA450 coated mesh; (**f**) Possible photocatalytic degradation mechanism of methylene blue by the TN450 coated mesh.

Since the pure TiO_2_ catalyst has no visible light photocatalytic activity, the excellent visible light catalytic property of TN450 coated mesh might be originated from the special chemical structure and element doping. Further study was carried out to confirm the hypothesis. The doped elements of TiO_2_ coated meshes were adjusted with the different calcination conditions. For example, the undoped TiO_2_ coated mesh was prepared by calcination of P(DVB-CH_2_N^+^Cl^−^)@TiO_2_ coated mesh under air atmosphere at 450 °C for 2 h to completely burn the polymer core and remove all C and N elements. The resultant mesh was labeled as TA450 coated mesh. On contrast, the C doped TiO_2_ coated mesh, labeled as TN550 coated mesh, was prepared by following a reported method which calcinated P(DVB-CH_2_N^+^Cl^−^)@TiO_2_ coated mesh at higher temperature of 550 °C in N_2_ atmosphere^66^. The surface elements of TA450 coated mesh and TN550 coated mesh were characterized by XPS as shown in Fig. 8a. For the sample TA450 coated mesh, the polymeric cores have been completely burned in air atmosphere without any signal of C and N elements in the full-scale XPS pattern. Hence, the surface of TA450 coated mesh is constituted by neat TiO_2_ hollow nanofibers. In the case of TN550 coated mesh, the peaks of N element are hardly found from the full-scale XPS pattern, while a strong peak of C 1*s* is existed. The result indicates the decomposition of Ti–O–N bond between 450 and 550 °C, thus C element sole doped TiO_2_ coated mesh is obtained.

The crystal structure of the two TiO_2_ coated meshes was characterized with XRD. As shown in Fig. 8b, the XRD patterns of TN550 coated mesh and TA450 coated mesh are very similar with the TN450 coated mesh. The diffraction peaks at 25.3° (101), 37.8° (004), 48.0° (200), 53.9° (105), 55.0° (211), 62.7° (204), 68.8° (116), 70.3° (220) and 75.0° (215) are attributed to the crystal planes of anatase (the ASTM Card, PDF#21-1272), indicating that the calcination conditions exhibit no changes in the anatase crystal form.

The visible light catalytic behavior of these two kinds of TiO_2_ coated meshes was studied, as shown in Fig. 8c. The residual content of methylene blue using TA450 coated mesh as photocatalyst is as high as 91.5 ± 2.2% after 120 min of illumination which is comparable to the self-degradation data of methylene blue under visible light, indicating that the undoped TiO_2_ coated mesh has no visible light catalytic ability. On contrast, the TN550 coated mesh exhibits a certain visible light catalytic activity with the residual content of photocatalyzed methylene blue at 35.5 ± 3.1% and the corresponding degradation efficiency at 64.5 ± 3.1% after 120 min of illumination. The results indicate that the C-doped TiO_2_ is mainly responsible for the visible light catalyst^61,73^. As shown in Fig. 8d, the first-order kinetic rate constant (*k*) is also inferred to compare the photocatalytic activities with the *k* at 0.0006, 0.0009, 0.0098, 0.0193 assigned to uncoated mesh, TA 450, TN550 and TN450 coated mesh, respectively. Obviously, the rate constants of TN450 coated mesh are ~ 20 and ~ 2 times as high as the TA 450 and TN550 coated mesh, respectively. The visible light catalytic ability of all TiO_2_ coated meshes can be arrayed in the order of C,N co-doped TiO_2_ coated mesh > C-doped TiO_2_ coated mesh > undoped TiO_2_ coated mesh, which is consistent with previous study of TiO_2_ based photocatalysts^65^. For further study of the photocatalytic degradation mechanism, the UV–Vis diffuse reflectance spectra of TiO_2_ coated meshes were characterized and shown in Fig. 8e. The TA450 (undoped TiO_2_) coated mesh exhibits an absorption peak in UV region, which indicate a good UV light catalytic activity. Compared with the TA450 coated mesh, both the TN550 (C-doped TiO_2_) coated mesh and the TN450 (C,N co-doped TiO_2_) coated mesh show an enhanced absorption peak in the visible light region (ranged from 200 to 800 nm). In particular, the absorption peak of C,N co-doped TiO_2_ coated mesh is significantly enhanced. Since all of the TiO_2_ on the three composited meshes are in anatase crystal form, it is reasonable to believe that the co-doped C and N elements play a synergistic effect on the enhancement of the visible light catalytic ability.

The TN450 coated mesh exhibits an even higher photocatalytic efficiency than the reported C,N co-doped TiO_2_ nanoparticles which could only degraded about 50% of methylene blue after 120 min of visible light illumination^64^. As summed by previous researchers, the enhancement in efficiency of TiO_2_ based photocatalysts was the result of their anatase crystallinity, high hydrophilicity, surface area and nano tubular morphology^74^. Moreover, some other parameters, such as porous or hollow structure^66,75,76^ and conductive carriers^60,62,77^, were beneficial to the photocatalytic efficiency of semiconductors. Based on the above discussion, a probable photocatalytic degradation mechanism of methylene blue with TN450 coated mesh is illustrated in Fig. 8f. Upon the visible light irradiation, a large number of electrons (e−) are stimulated on the conduction band (CB) of TN450 with holes (h+) generated on the valence band (VB) simultaneously. These photo-generated electron-hole pairs are quickly separated owe to the midgap states between CB and VB along with the hollow structures. Moreover, the electrons (e−) prefer to migrate toward conductive stainless-steel mesh and holes (h+) would remain on the surface of nanofibrous hollow TiO_2_, avoiding the recombination between electrons (e−) and holes (h+) and elongating the lifetime of charge carriers. Holes (h+) on TiO_2_ surface show strong oxidation which can indue the formation of hydroxyl radicals from water, following with the oxidation of methylene blue.

## Conclusion

The C,N co-doped TiO_2_ hollow nanofibers (TN450) coated stainless steel mesh was achieved via precipitate cationic polymerization, quaternarization and in-situ sol–gel process, following with calcination. The morphology was verified with SEM and TEM, showing well defined hollow nanofibers covering the mesh. The doped C and N elements are covalently bonded with the TiO_2_, confirmed from XPS pattern. The resultant TiO_2_ hollow nanofibers are anatase crystal form with a major interplanar spacing of 0.346 nm and a standard XRD pattern (PDF#21-1272). The coated mesh demonstrates superhydrophilic/underwater superoleophobic properties which can be wetted by water instantaneously but possess an underwater oil contact angle up to 159.3 ± 2.8°, 159.4 ± 1.5°, 162.6 ± 1.8° and 160.3 ± 3.4° to chloroform, toluene, *n*-heptane, and diesel, respectively. The specially wetted surface endows the composite mesh to separate the oil/water mixtures with a quite high proof pressure of oil and a high flux for water. Moreover, the TN450 coated mesh shows excellent degradation ability to methylene blue under both UV and visible light with the degradation efficiency at 99.5 ± 0.2% and 90.6 ± 2.4%, respectively, after 120 min irradiation. The photocatalytic activity is quite reliable with the degradation efficiency of methylene blue kept at around 97.8 ± 0.6% and 88.9 ± 1.2% after five cycles of photocatalysis experiments under UV and visible light, respectively. To understand the photocatalytic mechanism under visible light, the C sole doped TiO_2_ (TN550) coated mesh and undoped TiO_2_ (TA450) coated mesh were prepared through the controlled calcination. The degradation efficiency of methylene blue is only 8.5 ± 2.2% and 64.5 ± 3.1% photocatalyzed with TA450 coated mesh and TN550 coated mesh after 120 min of visible light illumination, respectively. The enhancement in the photocatalytic activity of TN450 coated mesh under visible light should be result from the C,N co-doping, the hollow nanofibrous structure and the conductive mesh framework synergistically. In general, the C,N co-doped TiO_2_ hollow nanofibers coated mesh exhibits credible oil/water separation capacity and excellent visible light photocatalytic performance, promising practical applications in complex wastewater treatment.

## Supplementary Information

Supplementary Information.

## Data Availability

All data generated or analysed during this study are included in this published article and its supplementary information file.
