# Supplementary material for: C,N co-doped TiO2 hollow nanofibers coated stainless steel meshes for oil/water separation and visible light-driven degradation of pollutants
Source: Sci Rep. 2023 Apr 7;13:5716. doi: 10.1038/s41598-023-28992-4 (PMC10082082; doi:10.1038/s41598-023-28992-4)
Supplement: Supplementary file 1 — Supplementary Information. [file 41598_2023_28992_MOESM1_ESM.docx]

**Electronic supplementary material**

**C,N co-doped TiO_2_ hollow nanofibers coated stainless steel meshes for oil/water separation and visible ​light-​driven degradation of pollutants**

Chunyu Wang^1,2^, Yingze Liu^2^, Hao Han^1^, Desheng Wang^1^, Jieyi Chen^3^, Renzhi Zhang^3^, Shixiang Zuo^3,4^, Chao Yao^3,4^, Jian Kang^1,*^ and Haoguan Gui^2,3,4,*^

^1^ State Key Laboratory of NBC Protection for Civilian, Beijing 102205, China

^2^ Department of Chemical Engineering, Tsinghua University, Beijing 100084, China

^3^ School of Petrochemical Engineering, Changzhou University, Changzhou 213164, China

^4^ Jiangsu Key Laboratory of Advanced Catalytic Materials and Technology, Advanced Catalysis and Green Manufacturing Collaborative Innovation Center, Changzhou University, Changzhou 213164, China

Corresponding Authors

[larance0130@163.com](mailto:larance0130@163.com) (J. Kang)

[guihaoguan@cczu.edu.cn](mailto:guihaoguan@cczu.edu.cn) (H. Gui)

**Additional Data:**

1. **Fig. S1.** Morphology of neat mesh and P(DVB-*co*-VBC) nanofibers coated mesh.
2. **Fig. S2.** EDX mapping image of P(DVB-CH_2_N^+^Cl^-^)@TiO_2_ nanofibers coated mesh and TN450 coated mesh.
3. **Fig. S3.** Morphology of PDVB-CH_2_N^+^Cl^-^ coated mesh calcinated at 450 ℃ for 2 h under nitrogen.
4. **Fig. S4.** UV-vis standard spectra of methylene blue solution


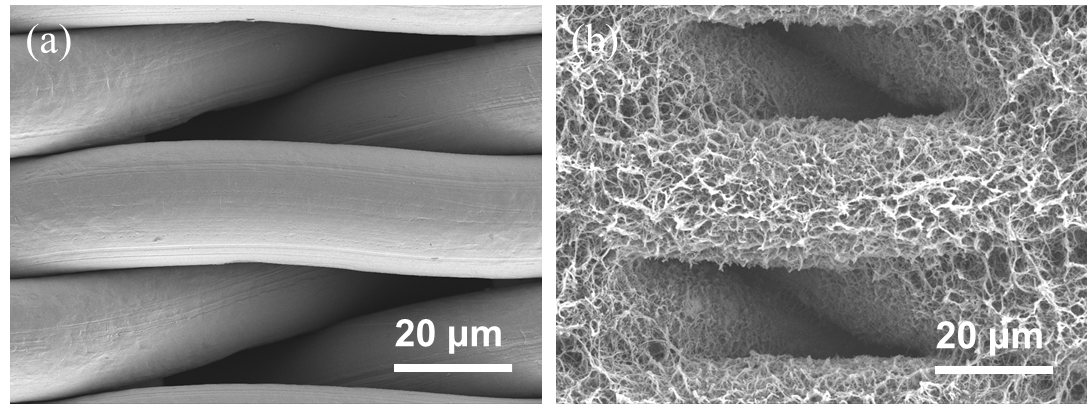


**Fig. S1.** (a) SEM image of pristine stainless steel mesh; (b) SEM image of P(DVB-*co*-VBC) coated stainless steel mesh.


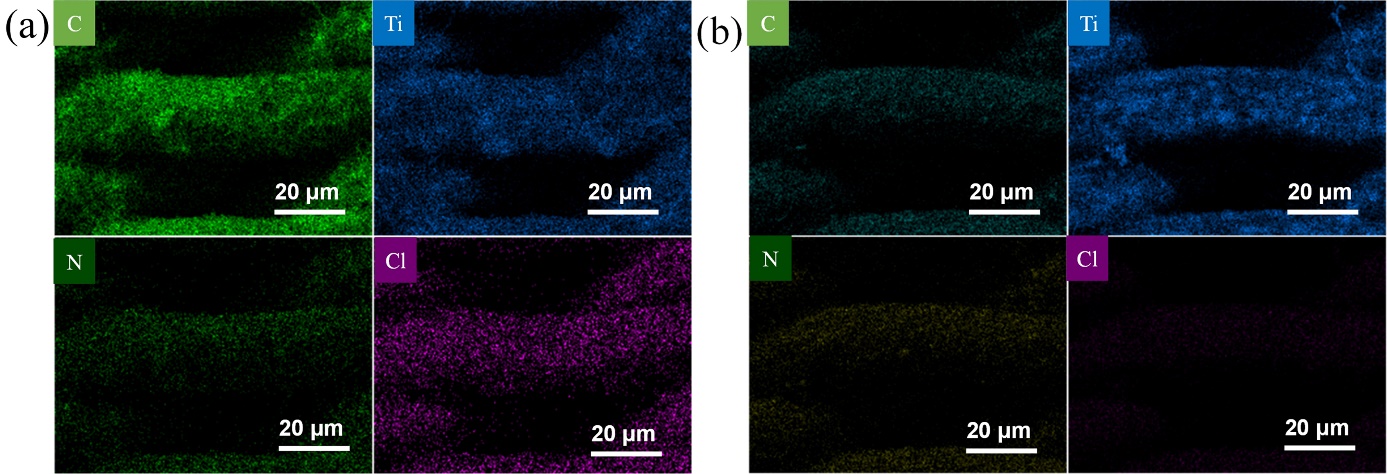


**Fig. S2.** (a) EDX mapping image of P(DVB-CH_2_N^+^Cl^-^)@TiO_2_ nanofibers coated mesh; (b) EDX mapping image of TN450 coated mesh.

**Fig. S3.** The SEM image of PDVB-CH_2_N^+^Cl^-^ coated mesh with calcination under nitrogen at 450 ℃ for 2 h. Inset: magnified SEM image.

**Fig. S4.** (a) The UV-Vis spectrum of methylene blue solution with different concentration; (b) absorbance versus methylene blue concentration.
